# Detection of self-paced reaching movement intention from EEG signals

**DOI:** 10.3389/fneng.2012.00013

**Published:** 2012-07-12

**Authors:** Eileen Lew, Ricardo Chavarriaga, Stefano Silvoni, José del R. Millán

**Affiliations:** ^1^Defitech Chair in Non-Invasive Brain-Machine Interface, Center for Neuroprosthetics, School of EngineeringEcole Polytechnique Fédérale de Lausanne, Switzerland; ^2^Laboratory of Robotics and Kinematics, IRRCSS Camillo Hospital FoundationVenice, Italy

**Keywords:** BCI, EEG, rehabilitation, self-paced protocol, stroke, voluntary movements

## Abstract

Future neuroprosthetic devices, in particular upper limb, will require decoding and executing not only the user's intended movement type, but also *when* the user intends to execute the movement. This work investigates the potential use of brain signals recorded non-invasively for detecting the time before a self-paced reaching movement is initiated which could contribute to the design of practical upper limb neuroprosthetics. In particular, we show the detection of self-paced reaching movement intention in single trials using the readiness potential, an electroencephalography (EEG) slow cortical potential (SCP) computed in a narrow frequency range (0.1–1 Hz). Our experiments with 12 human volunteers, two of them stroke subjects, yield high detection rates prior to the movement onset and low detection rates during the non-movement intention period. With the proposed approach, movement intention was detected around 500 ms before actual onset, which clearly matches previous literature on readiness potentials. Interestingly, the result obtained with one of the stroke subjects is coherent with those achieved in healthy subjects, with single-trial performance of up to 92% for the paretic arm. These results suggest that, apart from contributing to our understanding of voluntary motor control for designing more advanced neuroprostheses, our work could also have a direct impact on advancing robot-assisted neurorehabilitation.

## 1. Introduction

Human movements are usually volitional, where we spontaneously decide when to initiate it and commit to a particular course of action to accomplish a daily task (Haggard, 2008). This is the reason why uncovering the neural correlates of voluntary movement is important for implementing practical Brain Computer Interface (BCI) technology that people can use over long periods of time in a natural way. Current non-invasive BCI allows its user to deliver mental commands to a robot controller that transforms them into appropriate motor actions—e.g., left, right, and forward decoded from electroencephalography (EEG) signals while the user imagines different limb movements (Galán et al., 2008; Millán et al., 2009). However, most brain-actuated robots assume that the user wants to operate the neuroprosthesis in well-defined periods of time, in contrast to daily experiences of motor control, where movements are executed sporadically in a self-paced manner.

In this paper, we investigate the feasibility of detecting the intention to perform a reaching movement in single trials before actual execution from human EEG. Intention has been described as doing something purposefully (Schall, 2004). In this paper, we defined intention as the time of awareness of wanting to perform a reaching task. This definition is not to be confused with the work of Congedo et al. (2006), Gonzalez et al. (2006), and Bai et al. (2007) where movement intention was defined as the problem of classifying the intention to move the left hand or right hand. To study movement intention, we follow a self-paced paradigm where subjects can execute a reaching movement at any time they wish. This is a more natural and ecological experimental setup than the classical reaction task paradigm, where subjects perform movements in response to a cue.

A number of recent studies have found neural correlates of *when* subjects decide to initiate a movement. Through invasive methods, Fried et al. (2011) have reported progressive neuronal recruitment in the supplementary motor area (SMA) over 1500 ms before subjects made the decision to move. In another study with human electrocorticography (ECoG), Ball et al. (2009) reported the existence peri-movement activity as early as 200 ms before movement onset. With regard to non-invasive EEG studies, the earliest evidence of the neural correlates of voluntary movement intention was discovered by Kornhuber and Deecke (1965), who identified a slow, negative potential as early as 1.5 s before the execution of movement. This slow cortical potential (SCP) was initially named as Bereitschaftspotential. This readiness potential has two main components. The first one is a slow negative potential starting 1.5 s before voluntary movement. This negativity is more prominent over the central-medial scalp. The late component occurs 400 ms before movement, with a steeper slope over the contralateral primary motor area (Shibasaki and Hallett, 2006). The slow potentials originate in depolarizations of the apical dendritic tree in the upper cortical layers that are caused by synchronous firing, mainly from thalamocortical afferents, showing local excitatory mobilization for negative slow potentials (Birbaumer, 1999). The presence of this readiness potential was further analyzed in a series of famous studies by Libet et al. (1982, 1983) who showed that there is an unconscious preparatory brain activity that begins 1 s or more before movement, preceding the conscious awareness to act. Similar negativity components have been observed in patients with brain lesions (Deecke et al., 1987).

Nevertheless, being a SCP close to DC, the presence of the readiness potential in single trials seems to be elusive. Another EEG correlate of movement preparation and execution is the event-related desynchronization (ERD; Pfurtscheller and Lopes da Silva, 1999), a decrease in mu and beta power (8–30 Hz) over the contralateral primary motor cortex. Bai et al. (2011) showed that self-paced wrist extension movement onset can be detected on average 0.62± 0.25 s before actual movement from the analysis of ERD. Finally, Awwad Shiekh Hasan and Gan (2010, 2011) studied the EEG activity in the mu, beta and lower gamma bands (8–45 Hz) to detect movement onset also during self-paced wrist extension movements. They achieved good results, but with a poor temporal resolution (from 2 s before to 2 s after the movement).

Here we show for the first time the detection of self-paced reaching movement intention in single trials from the analysis of the readiness potential in 12 human volunteers, two of them stroke subjects. In this study, we used EEG signals filtered in a narrow frequency range of [0.1–1] Hz, which is reported to better capture anticipatory-related SCPs (Garipelli et al., 2011). We explicitly focus on the readiness potential for two reasons. Firstly, as mentioned above, it is a well-known correlate of voluntary movement intention. Secondly, it is a promising non-invasive method for localization of motor control after hemispheric lesions (Green, 2003), which could be useful for understanding motor functional improvements following rehabilitation.

In this respect, apart from contributing to our understanding of voluntary motor control and to the design of more advanced neuroprostheses, our work could also have a direct impact on advancing robot-assisted neurorehabilitation (Riener et al., 2005; Johnson, 2006). Indeed, robot-assisted therapy for stroke patients with moderate-to-severe upper-limb deficits has shown promising results in terms of improving motor functional recovery compared to traditional therapy (Kwakkel et al., 2008; Masiero et al., 2009; Staubli et al., 2009; Lo et al., 2010; Hogan and Krebs, 2011). Still this kind of neurorehabilitation therapy could be improved, as earlier detection of movement intention can minimize the delays in device activation and, thus, allow tighter coupling between the initial formation of the motor plan in the cortex and its execution at the periphery through movement-assisted devices, thus better promoting brain plasticity after stroke (Muralidharan et al., 2011). It is for this reason that, in one of the experiments, we have involved stroke patients in order to carry out a first feasibility study.

Single-trial classification of SCP has already been used in BCI, most notably by Birbaumer et al. (1999). Recently, Bradberry et al. (2010) showed the possibility of decoding arm trajectories from SCPs. Garipelli et al. (2009) has also analyzed the SCP for studying and classifying anticipatory behavior. Bai et al. (2007) explored the use of SCP, computed with a low-pass filter at 10 Hz, for classifying a right vs. left hand movement. In this work, the focus is on identifying the intention to execute a self-paced reaching action before the movement starts, irrespective of the movement direction or laterality. It is also worth noting that the readiness potentials have a similar shape to SCP associated to anticipatory behavior, in particular the contingent negative variation (Walter et al., 1964). However, as discussed in Rektor (2003), while both kinds of SCP are readily confounded in scalp recordings, more invasive techniques (Ikeda et al., 1994; Lamarche et al., 1995) or clever experimental designs (Ruchkin et al., 1986; Brunia and Damen, 1988) demonstrate differences.

The experiments and the proposed methods are detailed in section 2. In section 3, we report the experimental results where, in particular, we compare the effect of using manually and automatically selected channels. We also report on the classification of movement onset using electromyograph (EMG) signals as well as on the classification of non-movement intention period. Finally, we discuss the implications of our results in section 4.

## 2. Materials and methods

### 2.1. Experimental protocols

We have designed two experiments: (1) EEG recordings of free arm reaching movements to a target button from healthy subjects using only their dominant arms, and (2) EEG measurements of a high-precision arm reaching task from stroke patients and healthy subjects as a control group. The reason why, after the promising results achieved in the first experiment, we have run a second experiment with a small stroke cohort is to make a preliminary study on the feasibility to detect movement intention in single trials as a potential tool for rehabilitation. This experiment was done in a clinical setting. In this later experiment, subjects performed the task with both arms in order to analyze possible differences in performance between the paretic and healthy arms of patients.

#### 2.1.1. Experiment 1

Eight subjects (three female, age 29.33 ± 2.06) participated in the experiment. They were informed about the experimental procedures and gave their consent. All subjects were healthy with no known history of neurological abnormalities or musculo-skeletical disorders. Seven out of the eight subjects in this experiment were right-handed.

**Figure 1:**
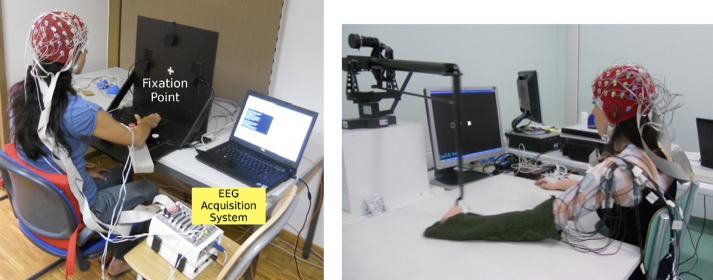
**Experimental setup for Experiment 1(left) and Experiment 2 (right)**.

The experimental workspace consisted of four targets (up, down, left, and right), which correspond to buttons on the horizontal plane located with respect to the mid-sagittal plane of the subjects as shown in Figure 1 (left). Despite the center-out reaching task, it is important to highlight that the decoding of reaching directions is not within the scope of this paper as, here, we are interested in studying the common initiation of movement, irrespective of the movement type. The dimension of the horizontal plane was 47.0 × 48.5 cm (length × width). The distance from the home position to each target positions was approximately 20 cm. The target and home position buttons were a disc with a diameter of 27 mm. The design of the targets and home position consisted of microswitch buttons with direct connection to the input trigger of the ActiveTwo (EEG recording device) USB2 receiver. The buttons act as the event marker for the movement onset. This design provides a high temporal resolution in marking the movement onset events (releasing the buttons from the center rest position). The recordings were conducted in a normal office environment, with people working and speaking around, to mimic as close as possible a realistic scenario.

Subjects were instructed to perform natural self-paced center-out and center-in arm reaching tasks with their dominant arm. They were asked to fixate their eyes on a cross in the middle of the vertical plane as shown in Figure 1 (left), thus minimizing eye movement-related artifacts in the recording. Each trial began with the subject placing their dominant hand on the center position. While at this position, subjects were asked to relax their hand, forearm, elbow, and arm in order not to induce any muscular tension which could possibly effect the outcome of the analysis. After 500 ms, an auditory cue informed the subject which target direction to reach. However, subjects were not supposed to react immediately (i.e., reaction task) or wait a fixed period of time (i.e., memory task) after the presentation of the cue. In contrast, they initiated the movement whenever they wish, but not before 2 s after the presentation of the auditory cue.

**Figure 2:**
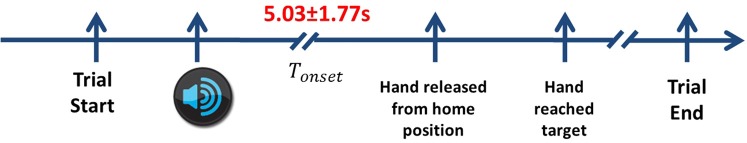
**The timeline of the experimental protocol.** Each trial starts when the subject places their hand on the center button. Next, the auditory cue informs the subject which direction to reach. After a delayed period of more than 2 s, he releases his hands from the home position and reaches towards the target. In order to complete the movement, the subject returns back to the home position before starting the next trial. Only center-out reaching periods are considered. The average *T*_*onset*_ across all subjects in Experiment 1 is 5.03 ± 1.77 s.

The role of the auditory cue was to ensure equal distribution of targets to be reached. There were a total of 200 trials recorded for each subject. Nevertheless, not all trials were kept for analysis. We discarded trials where the subject moved earlier than 2 s. We also removed trials if subjects reached to the wrong target. Finally, we discarded trials contaminated with strong artifacts or noise. After that, it remained an average of 188 trials across all subjects, where the average preparation time (*T*_*onset*_) is 5.03± 1.77 s as shown in Figure 2.

The design of this experiment allows voluntary initiation of movement by the subjects, in contrast with most cue-based reaction time task experimental protocols where there is a *go* cue that instructs the subject when to start the movement. It has been shown that the brain areas involved in a spontaneous task differ from those of an instructed task (Thut et al., 2000). In particular, they found longer lasting activity in the SMA during the spontaneous task and in the premotor area (PMA) during the instructed condition. Lu et al. (2011) also reported different brain areas responsible for cued and self-initiated movements.

The reaching task in this study is a form of unconstrained, multi-degree of freedom movement. Therefore, besides EEG, we also recorded EMG signals from the *musculus biceps branchii* (selection of location through trial and error before experiment) to monitor that there is no muscular activity during the preparation period. This signal was also used to determine the time onset of muscular activation with respect to the movement onset given by the experimental apparatus (i.e., microswitch at center position).

Before the experiment starts, subjects were asked to perform a calibration session where they have to move their eyes toward the targets, and to perform a 1 min natural eye blinking (Schlögl et al., 2007). This session was used to measure the effects of eye movements on the EEG signals (see section 2.2.2).

#### 2.1.2. Experiment 2

Four subjects, recorded at the San Camillo Hospital, Venice, Italy, participated in this experiment. There were two stroke patients and two healthy control subjects. All procedures were approved by the Ethics Committee of the San Camillo Hospital before experimentations. All subjects were informed about the experimental procedures and gave their consent.

Table 1 shows a summary of the subjects' particulars. All subjects were right-handed. Stroke subject **dpm** suffers from a left cerebellar hemorrhagic stroke, also commonly known as intracerebral bleed, where the ipsilateral body part is affected. Stroke subject **lg** suffers from a left nucleo-capsular stroke caused by lesion in a deeper brain structure, thus affecting the contralateral limb. Table 1 also reports the Fugl-Meyer Motor Assessment score for upper extremity (FMA-UE)—maximum score of 66—for both stroke subjects. Both patients had preserved tactile and proprioceptive sensibility of the arm with normal cognitive abilities at the time of admission to the hospital.

**Table 1:** **Details of subjects who participated in the Experiment 2**.

**Subject**	**Age**	**Medical condition**	**Paretic arm**	**Time since stroke**	**FMA-UE**	**Left hand MT**	**Right hand MT**
cg	25	Healthy	–	–	–	0.61 ± 0.19	0.58 ± 0.15
gc	26	Healthy	–	–	–	0.70 ± 0.17	0.66 ± 0.16
dpm	50	Stroke	left	55 days	56/66	**3.53 ± 1.63**	1.67 ± 0.73
lg	61	Stroke	right	658 days	43/66	1.59 ± 0.35	**2.34 ± 0.36**

MT refers to the time needed to complete a reaching movement. Values in bold show the performance with the paretic arm.

The subject was seated in front of a computer screen holding on to a haptic manipulandum (PHANTOM Premium 3.0/6DOF, Sensable Technologies) with her arm resting on the table as shown in Figure 1 (right). This experiment used a similar paradigm to the previous experiment. In contrast to the previous experiment, the reaching task was performed with both arms. The subjects were instructed to move a manipulandum that controls the position of a cursor (a green circle) on a computer screen. The rest position is the condition when the green circle remains inside the white box located in the middle of the screen. The task was to bring the cursor to one of the center-out target box. When the target was cued, the subject was asked to wait at least 2 s before initiating the movement. If he failed to do so, the subject had to move the cursor back to the rest position and wait for another 2 s before initiating the movement. The trial was discarded from analysis and repeated until the subject successfully fulfilled the requirement of 2 s delay period.

Subjects were asked to minimize their eye movements, in particular, before starting the arm movement. In this experiment, there was also a calibration session to record the baseline eye movement activity as in Experiment 1. The subject was asked to blink for 5 s, then, he had to look back and forth between the home position and the different targets as they appear on the screen where each target appeared five times. The recordings from the calibration session have been used for studying the effects of eye movements on the EEG channels (see section 2.2.2).

For each subject, we performed 3 recordings of 80 trials (targets are randomly cued), thus resulting in a total of 240 trials for each arm movement. After discarding early starts and artifacts, it remains an average of 229 trials for the left hand and 230 trials for the right hand across all subjects. For the stroke patients, the unaffected arm was tested first. The whole experiment lasted from 3 to 4 h, including the electrodes placement time. Each recording lasted from 6 to 15 min. Both stroke subjects were able to achieve the reaching task without much difficulty, but with longer average reaching time (as shown in Table 1) in comparison with the control subjects. Previous analysis with stroke subjects has reported that goal-directed arm movements are slower and more variable than healthy subjects' (Levin, 1996; Cirstea and Levin, 2000).

### 2.2. Methods

#### 2.2.1. EEG and EMG recordings

We acquired EEG potentials with a portable ActiveTwo measurement system from BioSemi (http://www.biosemi.com) using 64 electrodes arranged in the modified 10/20 International System. This system was also used to record the electrooculograph (EOG) signal. In Experiment 1, the Biosemi ActiveTwo measurement system was also used to record the EMG signals from the arm. As for the second experiment, the EMG signals were recorded with a Biopac System (http://www.biopac.com).

The signals were recorded at a sampling rate of 2048 Hz and downsampled to 256 Hz. To analyze EEG, we first applied the Common Average Referencing (CAR) procedure (Offner, 1950; Osselton, 1965), where, at each time step, the average potential over all the channels is subtracted from each channel. The re-referencing procedure removes the global background activity, keeping activity from local sources beneath the electrodes. The most intuitive implementation of a CAR is to use all the recorded channels (Bertrand et al., 1985). However, the EEG channels could be contaminated by noise, in particular by EOG and muscular artifacts, that may propagate to all other unaffected channels. In the next section we identify the EEG channels that are affected by EOG artifacts, which are the most prominent potential source of noise given the nature of the task and the frequency band to be analyzed. These channels are then removed from the analysis and, in particular, for computation of the CAR.

#### 2.2.2. Ocular artifacts

EOG signals were acquired from three electrodes positioned above the nasion, and below the outer canthi of the eyes (Schlögl et al., 2007). The bipolar EOG channels in the *left-central* and *central-right* positions were able to capture both the horizontal and the vertical EOG components.

Besides manual removal of noisy trials, we used a regression analysis method to assess the influence of EOG artifacts on each EEG channel (Schlögl et al., 2007). Briefly, channels having a large correlation with the EOG components are discarded from the montage before performing the CAR.

**Figure 3:**
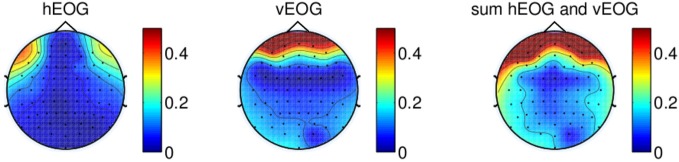
**Regression coefficients of EOG components plotted on a topographical map, showing the effect of eye movement on scalp electrodes using signal re-referenced with all 64-channels recorded from one of the subjects.** The rightmost figure shows the sum of the contributions of both vertical EOG and horizontal EOG.

Figure 3 illustrates the regression coefficients of the horizontal and vertical EOG components with the EEG channels computed in the calibration session for one of the subjects. The EEG signals are re-referenced using CAR with all 64 channels recorded from the experiment. This figure illustrates high contributions of eye movement artifacts in the frontal and temporal electrodes.

**Figure 4:**
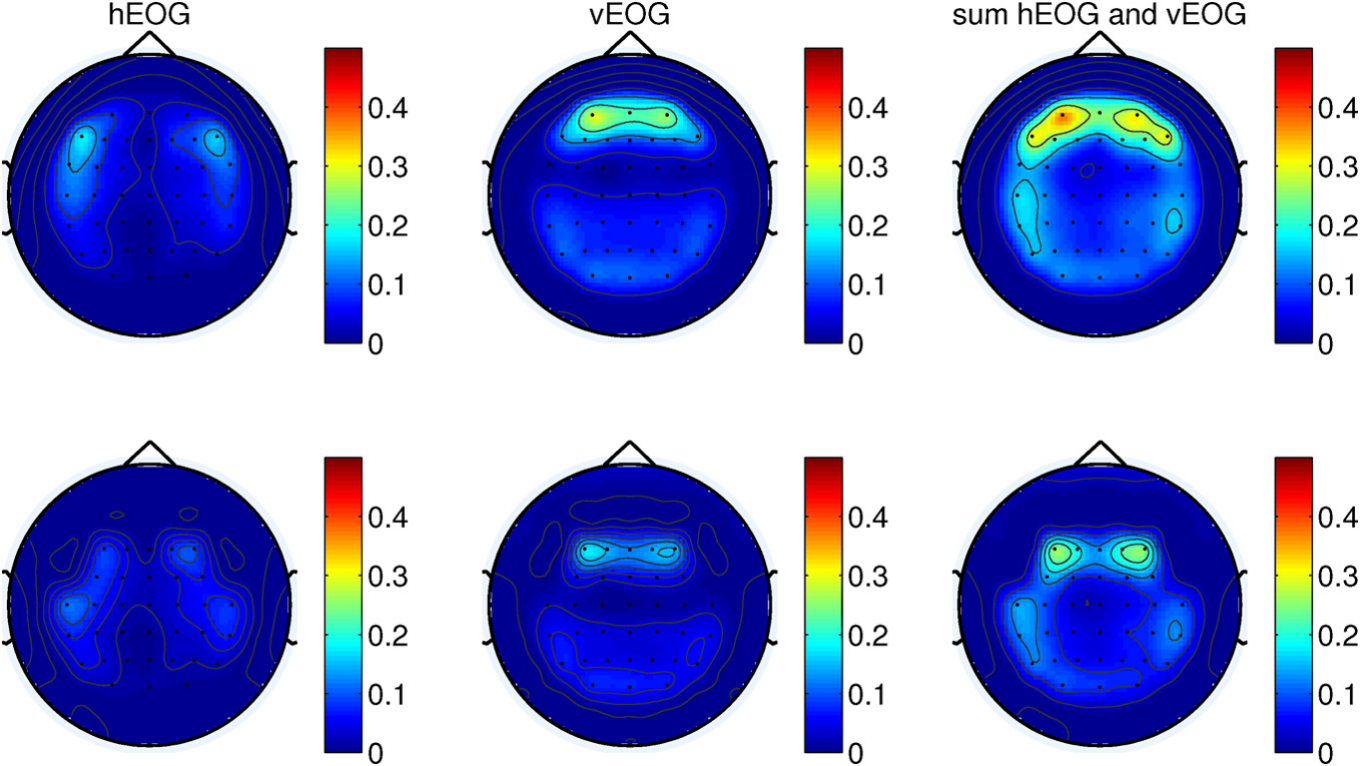
**(Top) The weights of EOG artifacts by re-referencing the signals with 41-channels and (bottom) 34-channels.** This figure shows the EOG coefficients from the calibration session of one of the subjects participating in the experiments.

We first remove the peripheral electrodes, filter the signals with CAR using the remaining 41 electrodes, and recomputed the coefficients. As shown in Figure 4 (top panel), the effects of vertical EOG is still high on the frontal electrodes. We further remove electrodes for which the coefficients were above 0.3. Thus, re-referencing the signals with a total of 34 channels can minimize the effects of eye movements on the scalp EEG as shown in Figure 4 (bottom).

**Figure 5:**
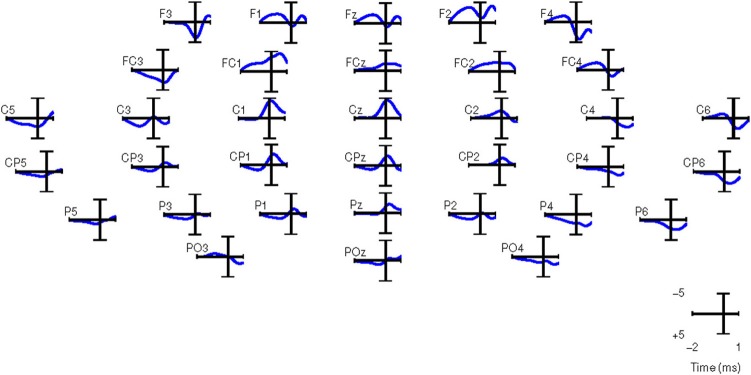
**Grand averages of SCPs for all the right-handed subjects participating in Experiment 1.** EEG signals are filtered between 0.1 and 1 Hz. *t* = 0 corresponds to the movement onset.

Similar results were obtained for all other subjects in both experiments. Therefore, in this paper, we performed the analysis with only 34 channels as shown in Figure 5.

#### 2.2.3. Pre-processing

The EEG signals were processed with a narrow band zero-phase non-causal IIR filter with cutoff frequencies of 0.1 and 1 Hz which has been reported to better capture anticipatory-related SCPs (Garipelli et al., 2011). The EOG signals were also preprocessed with the same method as the EEG signals.

EMG signals were acquired bipolarly over the *musculus biceps brachii* of the subject's arm, and high-pass-filtered with a Butterworth filter (8th order, cutoff of 50 Hz) to remove motion artifacts. The signals were then rectified, low-pass-filtered (8th order, cutoff of 20 Hz) and integrated over 25 ms to obtain envelopes of EMG activity (Cheung et al., 2009). The purpose of recording the EMG is to monitor that there is no muscular activity during the reaching preparation phase and to ensure that the movement intention detected is not due to the muscular activity of the arm through classification of the EMG activity (see section 3.3).

#### 2.2.4. Channel selection

We compare the classification performance using manually and automatically selected channels. In the first case, channels were selected on the basis of the grand-average SCP. In the case of automatic selection, the channels are ranked according to their discriminant power (DP; see below).

**Figure 6:**
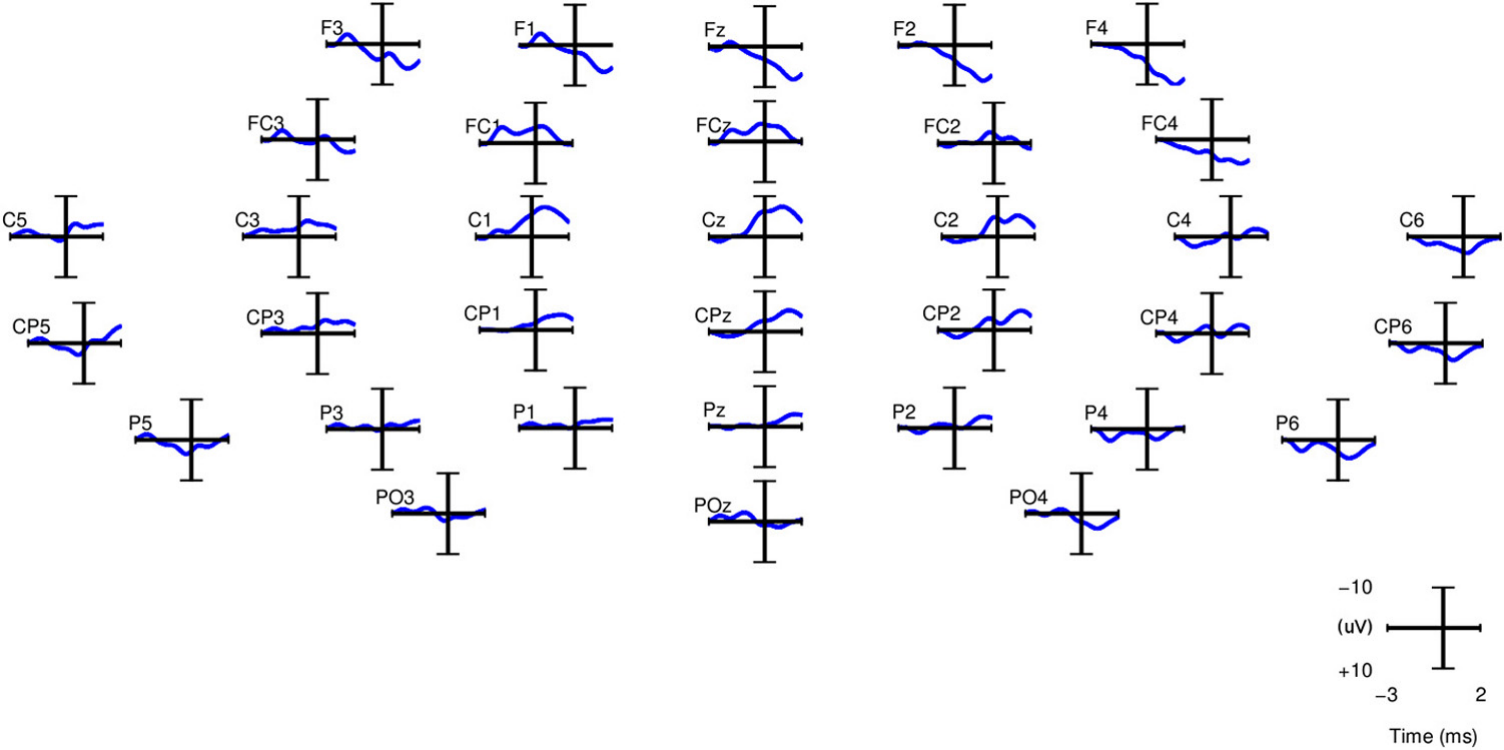
**Grand averages of SCPs, filtered between 0.1 and 1 Hz, for the paretic arm of stroke patient *lg* (right arm) from Experiment 2.** *t* = 0 corresponds to the movement onset.

For computing the grand averages of SCPs, each epoch was baseline corrected with the average activity between [2 2.25] s before the movement onset. Figure 5 shows the grand averages of SCPs over all right-handed subjects participating in Experiment 1 for each of the 34 channels. The SCPs in stroke subjects obtained from Experiment 2 exhibit a similar trend in the development of the negativity prior to the movement onset, as shown in Figure 6. However, the negativity peaked after 1 s of movement onset, when for control subjects the peak was roughly at movement onset (see also Figure 5). This is in agreement with Jankelowitz and Colebatch (2005), who recorded a larger and longer readiness potential when the stroke subjects moved the affected limb. The channels chosen manually for classification were C1, Cz, C2, CP1, CPz, CP2 as they exhibit prominent negative slopes in the grand average and are also consistent with previous literature (Kornhuber and Deecke, 1965; Libet et al., 1982).

**Figure 7:**
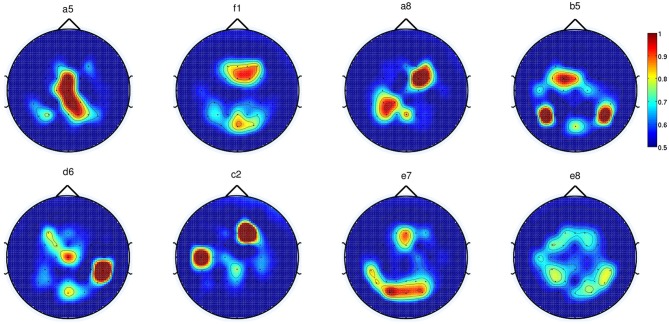
**Each topoplot shows the normalized discriminant power index of each channel for a single healthy subject in Experiment 1**.

Alternatively, we performed automatic channel selection using the Canonical Variant Analysis (CVA) (also commonly known as Multivariate Discriminant Analysis; Galán et al., 2007). This technique estimates the DP of each channel by comparing the movement preparation period to the non-movement related period. Figure 7 shows the DP value of each channel in the form of a topographic map for EEG signals in the frequency range [0.1–1] Hz for all eight subjects in Experiment 1. It is observed that the channels with high DP for movement preparation are different for each subject, suggesting subject-specific brain patterns in preparing reaching movement. With the exception of subject **a5** (where four out of six of the predefined channels match the most discriminant channels selected by CVA), the topoplots for the rest of the subjects showed high DP index in the frontal and parietal regions. According to Andersen and Cui (2009), the posterior parietal and frontal cortical areas are responsible for planning and decision making of movement intent. In this respect, it has also been reported that the frontal and parietal cortex region of the human brain carried considerable information to predict the outcome of a motor decision the subject had not yet consciously made (Soon et al., 2008). For subjects **f1**, **b5**, **e7,** and **e8**, CVA did not show any similarity with the pre-defined channels. Finally, we can observe that the topographic map for subject **d6**, the only left-handed subject in this study, showed high DP on the contralateral channels (right hemisphere).

**Figure 8:**
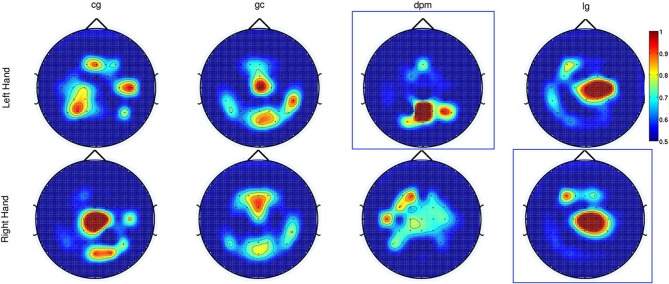
**The topographic maps show the normalized discriminant power index of each channel for the left and right hand for the control subjects (*cg* and *gc*) and the stroke subjects (*dpm* and *lg*).** Plots highlighted with a blue frame refers to the paretic arm of the patients.

As for Experiment 2, Figure 8 shows the DP value for each channel using the amplitudes of EEG signals filtered between [0.1–1] Hz for control subjects and stroke subjects (both left and right hand data). The regions showing the highest DP for healthy controls were similar to the observations in Experiment 1. Comparison between the pre-defined channels and the six most discriminant channels selected using the CVA method showed that four out of six channels were similar for stroke subject **lg** with data from both hands and for control subject **cg** only for the right hand. The topographic maps for stroke subject **lg** shows very consistent DP between left and right hand, and most importantly, the focus area is similar to the pre-defined channels set. As in Experiment 1, this central region is where the SCPs show high negativity prior to movement onset.

In section 3, we will show the performance differences between using pre-defined and CVA-selected channels.

#### 2.2.5. Classification

**Figure 9:**
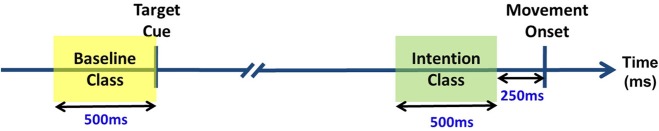
**Selected EEG samples to build the training set of the movement intention classifier**.

To detect the movement intention, we categorized the signals into two different time periods, namely the baseline period (idle period) and the movement preparation (active period). During the *idle period*, we assume that there is no on-going movement preparation activity. This period was taken 500 ms before the auditory target cue at each trial. The second part is the movement preparation period, which we termed as the *active period*, defined at [–750 –250] ms before the movement onset. Figure 9 depicts the selection of the EEG samples to train the movement intention classifier. Training data for the classifier consists of the baseline period (yellow box) as class *idle* and the movement preparation period (green box) as class *active*. As a classifier, we relied on Linear Discriminant Analysis (LDA), whose input was a vector with the EEG amplitude of the selected channels in a 500 ms window to capture the negative slope occurring 400 ms before start of movement. The EEG signals were subsampled from 256 to 8 Hz (four data points per window) before classification, resulting in a total of 24 features. To test the performance of our classifier, we used 500 ms windows shifted every 10 ms starting from 2500 ms before movement onset until 1000 ms after movement onset. We report below the results of a fivefold cross-validation. It is worth noting that the data used for feature selection is only based on the training data. Furthermore, we employed a cross-validation method which maintain the chronological order of the data (Millán, 2004; Bourdaud et al., 2008) which yields a better, and less optimistic, estimation of accuracy in comparison with the traditional method.

## 3. Results

### 3.1. Experiment 1

**Figure 10:**
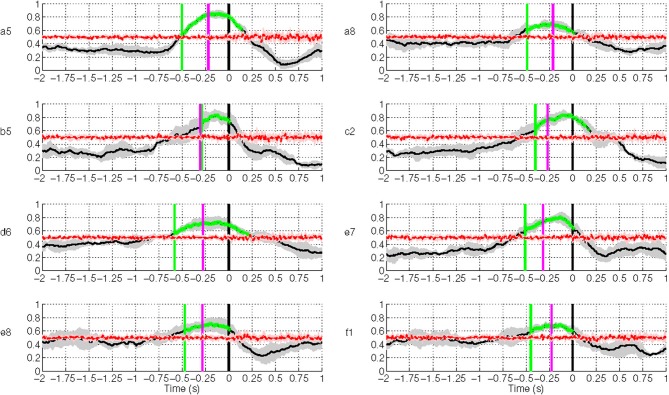
**Single trial performances of movement intention detection for all subjects in Experiment 1 using SCPs in the frequency range [0.1–1] Hz during the time interval (−2, 1) s with respect to the actual movement onset.** Y-axis of the plots represents the movement intention detection rate. The magenta line depicts the onset from arm muscular activation (−263 ± 40 ms on average across all subjects). The green line depicts the first time a group of five consecutive samples has a TPR significantly above chance level (*p* < 0.05), which is shown as a red line. The gray and red shaded regions bounding the performance curves indicates their standard deviation at each point. Note that the variance of the random performance is so small that the red shaded area is barely visible.

Figure 10 shows the results of movement intention detection where each plot represents the performance of a single subject in Experiment 1. Each plot reports the average *sensitivity* rate, or True Positive Rate (TPR), across the five test folds in the time window [−2, 1] s with respect to the actual movement onset. This can also be interpreted as the percentage of trials being detected as movement intention at time *t*. This time in the plot (X-axis) corresponds to the last sample of the analysis window analyzed by the classifier. The shaded region bounding the average TPR illustrates the standard deviation at each point. The magenta line refers to the onset of *biceps branchii* muscular activation. This is defined as the time when the EMG activity exceeds a threshold equal to μ + 3σ, where μ and σ are the mean and standard deviation of EMG signals of a one-second window after the target cue (Abbink et al., 1998). On average, all subjects exhibit an early arm muscular activity at 263 ± 40 ms. Similar EMG timing has been observed by Flanders (1991) and Hong et al. (1994) who studied the temporal patterns of muscles activation for unconstrained arm reaching movement in three dimensional space. The chance level line (in red) was calculated by shuffling the labels of the training data and performing 10 times fivefold cross validation. To test whether the sensitivity rate is significantly above the chance level with 95% confidence interval, we used the Wilcoxon rank sum test. The line in green depicts the first time a group of five consecutive samples has a true positive rate significantly above chance level (*p* < 0.05).

Movement intention can be detected above chance level across healthy subjects on average at 460 ± 85 ms before actual onset. The detection of movement intention is before arm muscular activation with the exception of subject **b5**. As reported in Table 2, the average maximum TPR was 0.76 ± 0.07, peaking on average 167 ms before movement onset. As we will see in section 3.3, although this peak performance is achieved slightly after EMG onset, using only EMG is a less reliable predictor for movement intention.

**Table 2:** **Maximum TPR for each subject and the time point (in ms) when this value is reached**.

**Subject ID**	**TPR**	**Time (ms)**
a5	0.85	−110
a8	0.70	−240
b5	0.83	−150
c2	0.84	−100
d6	0.72	−120
e7	0.80	−140
e8	0.71	−190
f1	0.69	−290
**Average ± std**	**0.76 ± 0.07**	**−167 ± 68**

**Figure 11:**
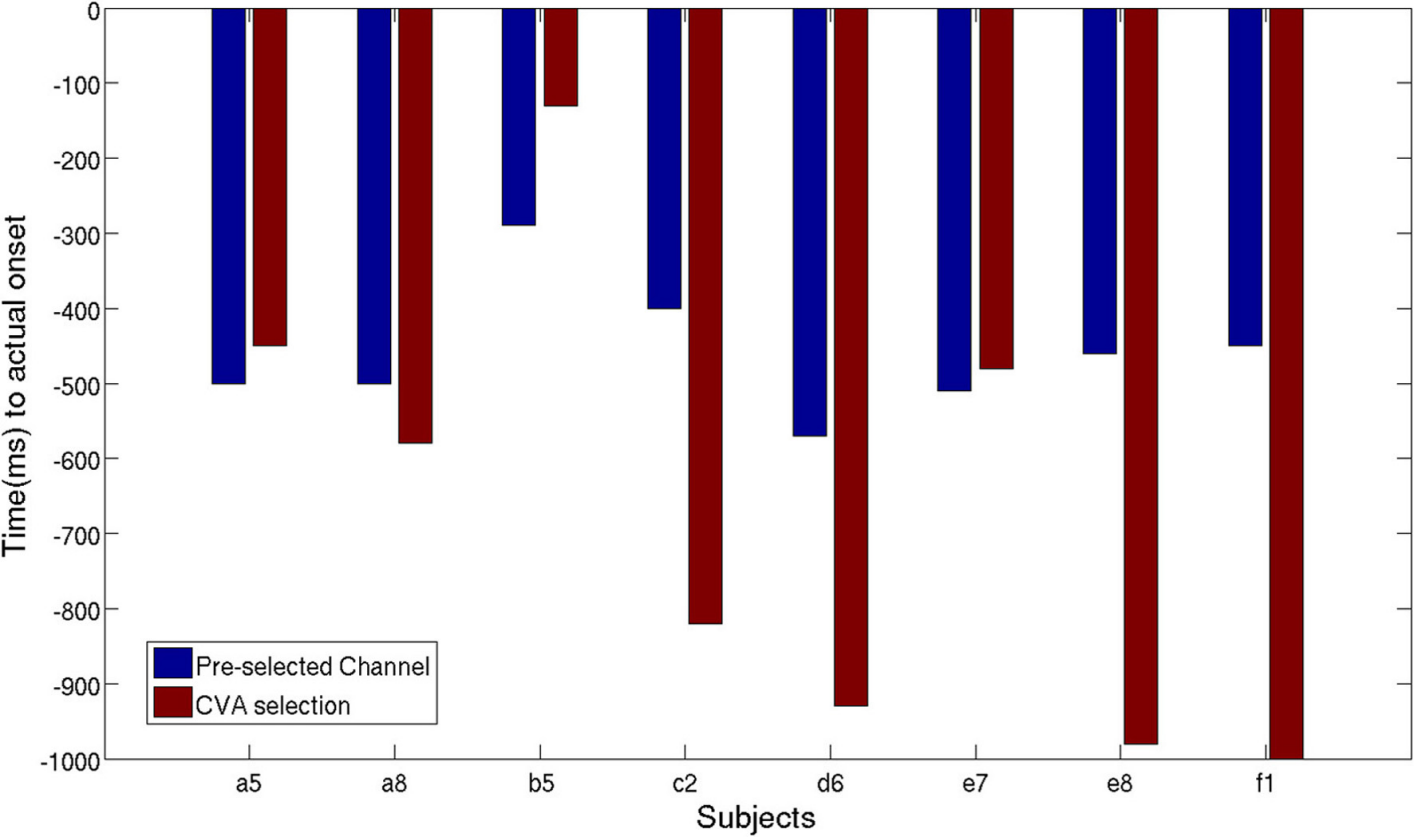
**Time of movement intention detection comparison between pre-selected channels set and best selected channel using CVA techniques**.

As described in section 2.2.4, we performed another analysis using the six best selected channels yielded by the CVA method. Figure 11 shows an earlier detection of movement intention with the features selected automatically, except for subject **a5**, **b5**, and **e7**. Unsurprisingly, for subject **a5** time differences are only 50 ms, since the CVA-selected channels highly overlap with the pre-selected set. The left-handed subject, **d6**, with high discriminability on the right lateral brain area, showed an earlier detection time of 360 ms using channels selected with CVA feature selection method. All subjects showed TPR above 70%, except for subject **e8** whose DP topographic map did not show a strong discriminative area.

### 3.2. Experiment 2

**Figure 12:**
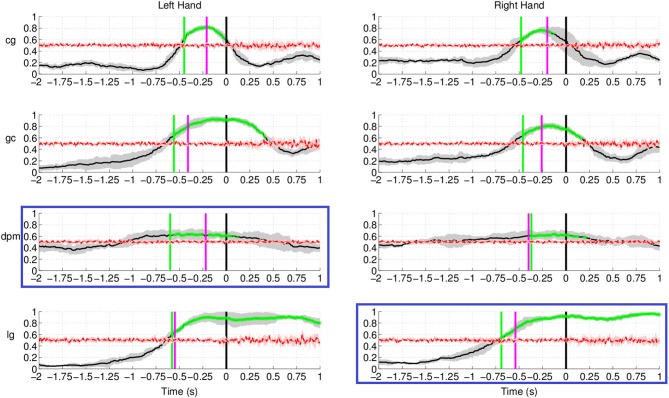
**Single trial performances of movement intention detection for all subjects in Experiment 2 (both left and right arm reaching movement) using SCPs in the frequency range [0.1–1] Hz during the time interval (−2, 1) s with respect to the actual movement onset.** This figure has a similar format to Figure 10. Plots highlighted with a blue frame refers to the paretic arm of the stroke subjects.

Figure 12 shows the results of movement intention detection for both the left and right hand reaching movement of all subjects (the upper graphs correspond to the healthy control subjects, followed by the two stroke patients). For all subjects, movement intention can be detected more than 400 ms before the recorded onset with their left hand and right hand. Movement intention can also be detected before the onset of EMG activity (magenta line, see previous section for details) for all subjects and conditions, except the non-paretic arm (right) of stroke subject **dpm**. The false positive rate prior to the detection of movement intention was also low (between 0.1 and 0.2 for both hands) for all subjects except stroke subject **dpm**. It is worthy to note that stroke subject **dpm** was a recent stroke patient (see Table 1) and, probably, the neural reorganization processes were still ongoing at the time of the experiment, which took place only 1.5 month after the stroke.

The result for stroke subject **lg** in Figure 12 (last row) exhibits a different performance curve as compared with the results of healthy controls. In particular, a high detection rate sustained up to 1 s after onset of movement. This difference could be due to the slower speed of reaching (c.f. Table 1) of stroke subject **lg** is 2.34 ± 0.36 s using his paretic arm compared to a faster speed of 0.5–0.6 s for healthy controls. The longer sustained high performance could also be due to the fact that the readiness potential of the affected limb in stroke subjects has higher amplitude over a longer period of time (Jankelowitz and Colebatch, 2005).

As reported in Table 3, the average maximum TPR obtained in this experiment was 0.81 for the left hand, peaking on average 140 ms before movement onset, while for the right hand the average maximum TPR is 0.79 at 162 ms before movement onset. It is interesting to note that for the two conditions where the peak performance is closer to movement onset (left hand of healthy subject **gc** and right hand of stroke subject **dpm**), performance stabilizes soon after EMG onset (in between −300 and −200 ms before movement onset) and slowly reaches its maximum value, which is 0.9 or higher. Altogether, these results are in agreement with those of Experiment 1 carried out with a larger set of subjects.

**Table 3:** **Maximum TPR for each subject and hand, and the time point (in ms) when the TPR reaches maximum value**.

**Subject ID**	**Left hand**		**Right hand**	
	**TPR**	**Time (ms)**	**TPR**	**Time (ms)**
cg	0.84	−200	0.79	−310
gc	0.90	−30	0.80	−140
dpm	0.66	−100	0.63	−140
lg	0.85	−230	0.92	−60
**Average ± std**	**0.81 ± 0.11**	**−140 ± 92**	**0.79 ± 0.12**	**−162 ± 105**

**Figure 13:**
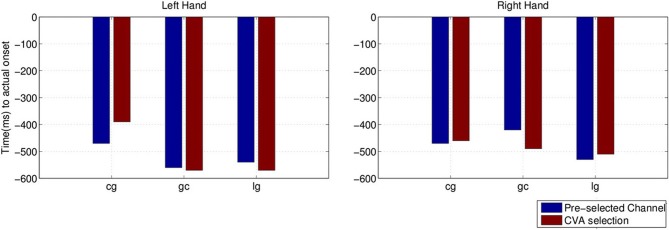
**Comparison of detected movement intention when TPR is above chance level (*p* < 0.05) between using pre-selected channel set and best selected channel from the data using CVA technique**.

We also compared the earliest time of onset detection using either the pre-selected channels or the channels chosen with the CVA data driven approach. Stroke subject **dpm** was excluded from the comparison because of the random results, to avoid misleading conclusion from the earlier detected intention. Figure 13 shows the earliest time when movement intention was detected for the two control subjects and stroke subject **lg**. Differences in time and performance between the two approaches are not significant.

### 3.3. Effects of muscular artifacts

In this section, we are interested in studying how and when movement intention can be detected from the arm muscular activity. To model the movement class, we take the window ended at 0 s (between −500 and 0 ms) because the grand averages of EMG activity showed no movement on average 250 ms before the movement onset.

**Figure 14:**
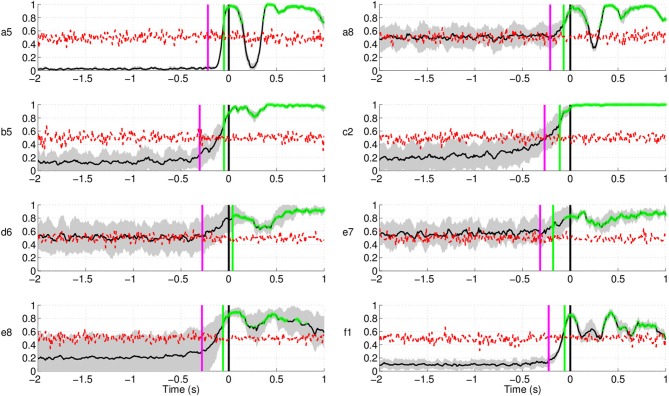
**Single trial detection of movement intention from EMG activity for all subjects in Experiment 1.** Y-axis of the plots represents movement intention detection rate.

Figure 14 shows the EMG classification results using the same technique as in the case of EEG for the subjects in Experiment 1. The results show that movement intention can be detected from EMG activity at a time point close to the actual onset derived from the button release. Interestingly, the EMG classifier detects movement intention *after* the thresholding method (magenta line in Figure 14) and *significantly later* than the EEG classifier. We can thus conclude that detection of movement intention from EEG signals is not due to muscular artifacts.

**Figure 15:**
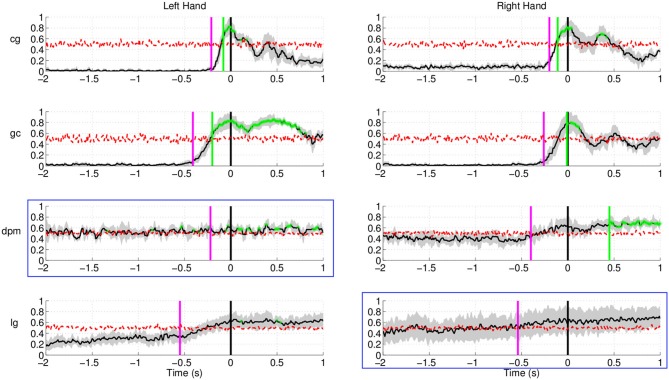
**Single trial detection of movement intention from EMG activity for all subjects and hands in Experiment 2.** Plots highlighted with a blue frame refers to the paretic arm of the patients. Y-axis of the plots represents movement intention detection rate.

Similarly, in Experiment 2 (Figure 15) movement intention can be detected significantly above chance level *only after* the time obtained from the thresholding method (magenta line in Figure 15) for the control subjects, which is in agreement with the results from Experiment 1. The EMG signal classification for both stroke patients, however, yielded random level classification, showing that these signals cannot be used to detect reliably movement intention or onset. Further analysis of other muscles, such as triceps and deltoid, yielded similar results. The reason for this is that, given the precision and spatial accuracy required in this task, agonist and antagonist muscles are activated synergistically to achieve a fine control of the forearm.

Altogether, these results show that detection of movement intention from EEG signals occurs before the muscular activation, showing high probability that preparation for movement happens before the peripheral system reacts and this information could be exploit for detecting the intent to move. This result is also in line with the behavioral study of Libet et al. (1983), where participants in the experiment reported the conscious intention to act 206 ms before the onset of muscle activity.

### 3.4. Analysis of the non-movement intention period

**Figure 16:**
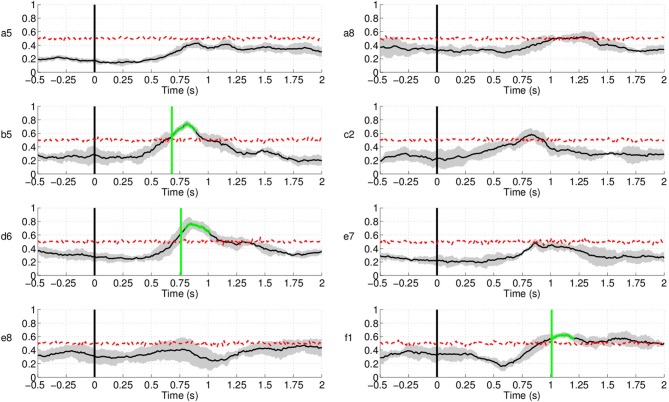
**Detection of movement intention during the non-movement intention period for all subjects in Experiment 1.** Time 0 s refers to the delivery of the auditory target cue. Y-axis of the plots represents movement intention detection rate.

Up to now, we have studied the performance of the EEG classifier to detect movement intention during the preparation period. The results show a quite high *sensitivity* rate of the EEG decoder. In this section, we analyze the *specificity* of such an EEG classifier. To do so, we examine the performance of the proposed method during the non-movement intention period—i.e., the time where subjects should not engage in preparing the movement. Figure 16 shows the rate of trials detected as movement intention during such a period lasting from –1000 ms before the auditory target cue until 2000 ms afterwards for all subjects in Experiment 1. Since windows for classification are 500 ms long, the first decision point is at −500 ms before the target cue. Interestingly, the detection of movement intention remains significantly below random level over the whole period preceding the target cue. And, remarkably, this is also the case during the first 2 s after the target cue (when the subjects should not move) for five out of eight subjects. The remaining three subjects (**b5**, **d6**, and **f1**) reached detection rates significantly above random, but only for a short period of time (less than 250 ms for all three subjects, starting at 750 ms after the target cue for **b5** and **d6**, and at 1000 ms for **f1**) before they decreased rapidly below chance level again. This may reflect some form of movement preparation after the subjects were informed of the target that they suppressed afterwards.

**Figure 17:**
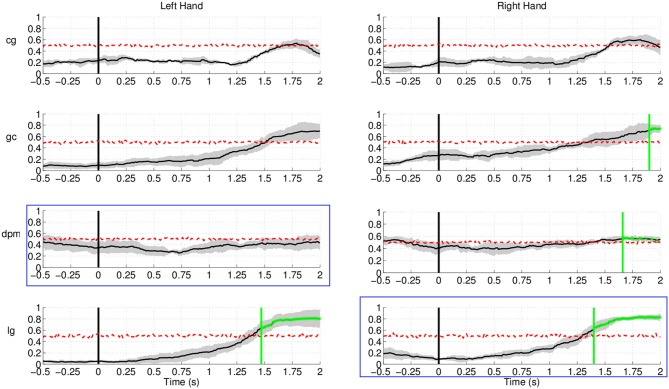
**Detection of movement intention during the non-movement intention period for all subjects and hands in Experiment 2.** Time 0 s refers to the delivery of the visual target cue. Plots highlighted with a blue frame refers to the paretic arm of the patients. Y-axis of the plots represents movement intention detection rate.

Regarding Experiment 2, Figure 17 shows lower (false) detection rates, at approximately 10%, for both control subjects and one of the stroke subject, **lg**. Detection rates started rising approximately 1.5 s after the target cue, with the exception of stroke subject **dpm**, who showed constant random level performance throughout the entire period. A plausible explanation for this increase is that, in this experiment, subjects had a large number of trials where the movement onset was between 2 and 3 s after the target cue, in particular stroke subject **lg**.

As a conclusion, our approach demonstrates to have a high sensitivity and a reasonably good specificity (below random detection level during the non-movement period) to allow robust single trial detection of movement intention from human EEG.

## 4. Discussion

**Figure 18:**
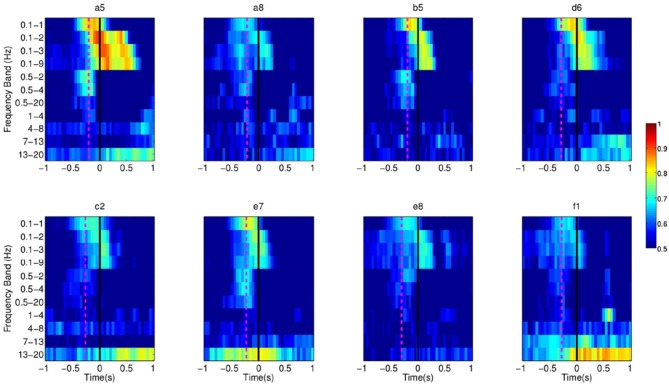
**Each pixel refers to the single trial performance of movement intention detection for all subjects in Experiment 1 using signals filtered in various frequency ranges (Y-axis).** The dotted line in magenta refers to the EMG activation for each subject.

Our experiments, involving healthy subjects and stroke subjects, demonstrate successful single-trial detection of movement intention from EEG prior to the actual movement in a self-paced reaching protocol. In particular, we show the detection of self-paced reaching movement intention in single trials from the analysis of the readiness potential, an EEG slow potentials that we compute in a narrow frequency range between 0.1 and 1 Hz. In these experiments, SCPs seem to carry most of the relevant information for the detection of movement intention as performance is higher than other frequency bands, as shown in Figure 18. In future work we will explore whether coherence among different EEG frequencies and channels, reflecting the rather complex brain network involved in this task, could offer further insight into the underlying mechanism of self-paced movement preparation and improve the performance of the detection.

Our SCP approach yields high detection rates close to the movement onset (sensitivity) and below random detection level during the non-movement period (specificity). Also, movement intention was detected around 500 ms before actual onset, in agreement with previous studies on readiness potentials using grand average activity (Kornhuber and Deecke, 1965; Libet et al., 1982). To further increase the performance of our method, in particular its specificity, we could improve our experimental protocol in order to better model the non-movement intention period. It would suffice to incorporate null trials (i.e., no movement trials). We will also explore the use of an evidence accumulation framework (Perdikis et al., 2011) that have proven beneficial in BCI as it only issues commands with high probability of confidence levels.

Previous works on movement onset have focused on hand/wrist flexion only (Awwad Shiekh Hasan and Gan, 2010, 2011; Bai et al., 2011), without reaching to a definite goal. Cortical activity is different in both cases. Readiness potentials, and their associated topography, have been found to be modulated by the consequence of movement, complexity of the movement, level of skill, sequence of hand movements; as well as the part of the body performing the movement, force, speed, and precision of a movement (Lang, 2003). In particular, Simonetta et al. (1991) reported larger amplitudes of the readiness potential in sequential motor tasks than in simple movements. There is also a larger late BP in self-paced movement of the proximal than the distal part of the upper extremities (Jankelowitz and Colebatch, 2002). Finally, different studies have reported that the attentional level has an influence on the neural correlates of movement onset. Libet et al. (1982) showed differences in the shape of the readiness potential depending on subjects' strategies, either involvement of general preplanning to act in the near future or direct movement when subjects were aware of the need to move. The former showed earlier onset (about 1 s). Keller and Heckhausen (1990) compared the readiness potentials between consciously and unconsciously performed motor actions, and found larger amplitudes in Cz, FCz, and Fz with consciously performed movements.

A previous study with four stroke subjects (Muralidharan et al., 2011) reported that attempted finger extension could be detected in stroke subjects with accuracy rates varying across subjects with a maximum true positive rate of 70% through combinations of PSD in the range of [2–30] Hz. These results, however, were obtained in a reaction task paradigm where subjects performed the movement or relaxed in response to a cue. In our study, the average maximum true positive rate was 0.81± 0.11 across both groups, controls and stroke subjects. The performance for one of the stroke subjects, **dpm**, was slightly above random, with maximum TPR of 0.66, while for another stroke subject, **lg**, the maximum TPR was 0.92 for reaching trials executed with his paretic hand. Although promising, the results achieved with stroke patients can only be taken as a preliminary feasibility study because of the limited number of subjects involved in the study. Nevertheless, it is worth noting that one of the patients achieved similar performance to the healthy subjects with the paretic arm.

In this work we have explored the use of EEG readiness potentials to decode a key aspect of voluntary movement behavior, namely self-paced onset. But it could also be related to another critical aspects of voluntary behavior, in particular volitional inhibition—stopping or changing a planned motor action that is not any more appropriate to the current context. In fact, Chen et al. (2010) have found that SMA is involved in both, movement preparation and movement inhibition. It would be interesting to detect the onset of an inhibitory process in a reaching version of the countermanding paradigm proposed by Mirabella et al. (2006, 2008, 2011). In particular, Mirabella et al. (2011) shows the existence of neurons in the dorsal premotor cortex exhibiting a pattern of activity compatible with the control of reaching arm movement initiation and suppression, thus suggesting that motor cortices are the final target of the inhibitory command elaborated elsewhere. The identification of the inhibitory process onset, in conjunction with detection of voluntary self-paced movement onset, may lead to more efficient and natural neuroprosthetics as well as more effective post-stroke motor rehabilitation training.

Detection of voluntary movement intention prior to its actual execution is a new capability that may advance the current state of the art in BCI and neurorehabilitation. For motor recovery, triggering the robotic-assistive device before EMG activation can largely improve the outcome of therapy (Muralidharan et al., 2011). In this case, decoding readiness potentials suits naturally in the design of goal-directed protocols where patients need to execute purposeful actions, which have been shown to produce significantly smoother, faster, and more forceful movement than repetitive routine movement (Trombly and Wu, 1999). In the case of motor substitution, it will provide a natural signal to enable usual brain control of wheelchairs and upper limb neuroprostheses while blocking their operation until the subject wishes to do so. The results reported here are certainly encouraging and can be extended in a couple of ways for its practical application in a neuroprosthesis. Future work will be devoted to test the proposed method in an online implementation and perform the analysis with more disabled users. In particular, subjects could learn to control a robotic arm. It will be interesting to analyze the learning effects and the stability of the signals during such closed-loop real-time control applications. Regarding neurorehabilitation, as discussed in the Introduction, it would be extremely exciting to try our approach in combination with rehabilitation robotics for motor recovery of spinal cord injury and stroke patients.

### Conflict of interest statement

The authors declare that the research was conducted in the absence of any commercial or financial relationships that could be construed as a potential conflict of interest.
